# Perspectives of public health organizations partnering with refugee, immigrant, and migrant communities for comprehensive COVID-19 case investigation and contact tracing

**DOI:** 10.3389/fpubh.2023.1218306

**Published:** 2023-09-05

**Authors:** Elizabeth Dawson-Hahn, Windy Fredkove, Sayyeda Karim, Farah Mohamed, Seja Abudiab, Diego de Acosta, Sabrina Ebengho, Yesenia Garcia, Sarah Hoffman, Megan Keaveney, Erin Mann, Christine Thomas, Kimberly Yu, Katherine Yun

**Affiliations:** ^1^Department of Pediatrics, University of Washington, Seattle, WA, United States; ^2^National Resource Center for Refugees, Immigrants and Migrants (NRC-RIM), University of Minnesota, Minneapolis, MN, United States; ^3^School of Nursing, University of Minnesota, Minneapolis, MN, United States; ^4^Bille Consulting, Seattle, WA, United States; ^5^Seattle Children’s Research Division, Seattle, WA, United States; ^6^Centers for Disease Control and Prevention, Atlanta, GA, United States; ^7^Department of Medicine, University of Minnesota, Minneapolis, MN, United States; ^8^Perelman School of Medicine, University of Pennsylvania, Philadelphia, PA, United States

**Keywords:** case investigation and contact tracing, refugees, immigrants, migrants, qualitative

## Abstract

**Objectives:**

To understand public health organizations’ experiences providing comprehensive COVID-19 case investigation and contact tracing, and related promising practices with refugee, immigrant and migrant communities.

**Methods:**

We interviewed public health professionals (September 2020 to February 2021) from local and state health departments using a geographically stratified, purposive sampling approach. A multidisciplinary team at the National Resource Center for Refugees, Immigrants and Migrants (NRC-RIM) conducted a thematic analysis of the data.

**Results:**

Six themes were identified: understanding community and public health context, cultivating relationships, ensuring linguistic and cultural concordance, communicating intentionally, evolving response, and implementing equity. The interconnection of themes and promising practices is explored.

**Conclusion:**

As public health continues to learn from and build upon COVID-19 response experiences, the thematic findings and potential promising practices identified in this project may foster proactive, community-engaged solutions for public health, and other organizations working and partnering with refugee, immigrant, and migrant communities. Implementing these findings with COVID-19 into current and future public health crisis responses may improve public health, collaborations with refugee, immigrant, and migrant communities, and staff wellbeing.

## 1. Introduction

Globally about one in seven people have experienced migration (1). The COVID-19 pandemic has had a disproportionate impact for refugee, immigrant, and migrant (RIM) communities in high-income countries outside the United States and in regional studies within the United States. People in RIM communities may have had limited access to testing (2, 3), higher risk of exposure (4, 5), higher risk of infection (3, 4, 6) and hospitalization (7), and limited access to vaccination (8, 9).

Early in the COVID-19 pandemic, the United States Centers for Disease Control and Prevention identified universal case investigation and contact tracing (CICT) as a core public health measure to interrupt transmission (10). In the context of other infectious diseases, CICT has been challenging when programs have limited capacity to interview people who speak non-dominant languages and do not have dominant-language proficiency (11). Ensuring staffing capacity in languages other than the dominant language, and recognizing community assets are both key to successful CICT with RIM communities (12). Sufficient public health capacity, staff training, community engagement, and education about the role of CICT are needed for CICT to be an effective tool for COVID-19 mitigation (13, 14).

Though there is an expansion of literature describing multiple aspects of the COVID-19 pandemic response in RIM communities, understanding the perspective of public health organizations about barriers and facilitators of effective and community-centered CICT and examples of promising practices are needed. The National Resource Center for Refugees, Immigrants, and Migrants (NRC-RIM) (15) sought to understand the perspective of public health, health (16), and community organizations (17) on CICT through a series of qualitative interviews. This manuscript describes the perspectives of professionals working within public health organizations on facilitators and barriers to CICT, and links them to promising practices of comprehensive CICT with RIM communities.

## 2. Methods

We conducted qualitative interviews with two goals: (1) to understand the perspective of public health organizations engaged in comprehensive CICT and (2) to identify promising practices implemented by public health partners working with RIM communities during the COVID-19 response. We define comprehensive CICT as the continuum of engagement with public health organizations to support people who were infected with or exposed to COVID-19, including culturally responsive strategies such as health education and communication, testing, case investigation, contact tracing, quarantine and isolation, health monitoring, and resource provision.

### 2.1. Interviewees

We interviewed public health professionals from local and state health departments across the United States using a geographically stratified, purposive sample approach across the Health and Human Services regions. Eligible interviewees were involved in some component of the COVID-19 CICT continuum. We asked interviewees to describe their professional perspective working within a public health organization including reflecting on programmatic and organizational approaches to CICT. We refer to dominant and non-dominant language in the introduction since differences in language between public health organizations and members of our communities is universal. We will, however, be focusing on English specifically in this manuscript since the interviews were conducted within the United States and focused on the United States public health response to COVID-19 at local and state health departments. Interviewees were identified through a network of public health practitioners and health care providers, the North American Refugee Health Service Providers listserv, and the Association of Refugee Health Coordinators listservs. The perspectives of professionals within health systems and community experts/organizations are reported elsewhere (16, 17).

### 2.2. Data collection and data analysis

Interviews were conducted from September 2020 to February 2021. We developed a semi-structured interview guide (Supplementary Table 1) with input from public health professionals and extant CICT literature. Demographics were collected via REDcap (18, 19) for each interviewee. Interviews were conducted in English, audio recorded, transcribed by a professional transcription service and analyzed in Dedoose version 9.0.107 (20).

Organizational level descriptive statistics were computed. A multidisciplinary team followed the phases of thematic analysis outlined by Braun and Clarke (21). Three team members participated in identifying patterns in the data. The first five transcripts were independently coded by two coders, discussed and reconciled as needed, guiding the iterative codebook development. Subsequently, one transcript was selected for review by two team members independently, followed up by a group discussion. The remaining 16 interview transcripts were coded by one team member (one team member coded 5 and the other team member coded 11). The codes were reviewed and patterns in the data were discussed during weekly team meetings. Upon completion of coding, themes were identified from observed data patterning, iterative thematic mapping, summaries and revisions, discussions and naming. Members of the broader team were intermittently involved in reflexive discussions about analytic team memos and thematic interpretations (21, 22).

We identified promising practice examples within the interviews by team consensus and they were shared on the NRC-RIM website for rapid dissemination as the team learned about them (15). The majority of the promising practices on the NRC-RIM website were identified from this set of interviews with public health organizations, with interviews with health systems (16) and community organizations (17), from review of the media or shared by partner organizations. After the thematic analysis, two team members identified promising practices from the NRC-RIM website to link to the themes in this analysis in order to provide concrete examples of promising practices. First, promising practices that were from the interviews in this data set were selected. Then if a promising practice was not available from this data set that aligned with the theme then through consensus three team members identified another example from the NRC-RIM website.

## 3. Results

### 3.1. Interviewee characteristics

We conducted 21, 45–60-min interviews with a total of 33 public health professionals, some interviews included more than one person (Table 1). Interviewees’ roles included: State Refugee Health Coordinators and Program Leads, Public Health Nurses, Epidemiologists, Program Leads for CICT, Medical Directors of Public Health Clinics, and City/County Health Officers.

**Table 1:** Characteristics of participating organizations (*N* = 21) engaged in comprehensive COVID-19 case investigation and contact tracing with refugee, immigrant, and migrant communities from the United States HHS Regions from September 2020 to February 2021.

	Public health organizations (*N* = 21)
Total number of interviewees^*^	33
Location, by HHS region	
1 or 2	2
3 or 4	7
5 or 6	4
7 or 8	3
9 or 10	5
Organizational level^**^	
Local (City/County)	8
State	13
Regional	0
Organizational type	
Public health	21
Immigrant-specific organization^***^	11
Populations served^****^	
Refugees	9
Migrant workers	8
Other immigrants	9
Interview completed after first COVID vaccine EUA^*****^	6

* Many organizations requested group interviews with two or more staff members.

** Organization level was categorized as local (e.g., city or county) even if part of a state-wide, regional, or national group when the operational unit that participated in the interview was focused on a local area. For example, an interview focusing on an FQHC’s city-wide programming would be categorized as “local” even if the FQHC was part of a state-wide FQHC network.

*** We categorized organizations as “refugee, immigrant, migrant-specific” if the organization as a whole or the operational unit within the organization that participated in the interview (e.g., a state refugee health program within a Department of Public Health) focuses specifically on RIM communities.

**** Many organizations work with more than one population.

***** December 11, 2020.

### 3.2. Themes and promising practices

Six themes were identified. Interview excerpts representing each of the six themes from this analysis are displayed in Table 2. In addition to the data examples supporting each theme, Table 2 links promising practices identified by interviewees from this data set and NRC-RIM website with the thematic analysis to provide concrete examples of these themes in action.

**Table 2:** Select comprehensive CICT promising practice examples and supporting data excerpts by primary theme^*^ (interview data from across the United States HHS Regions from September 2020 to February 2021).

Promising practices	Supporting thematic analysis excerpts
	Theme: understanding community & public health context
Working toward equitable language access	*The other group I work with is on communication, so we have been analyzing monthly surveys for the Municipality of* [city name] *since the beginning of the pandemic, and one of the things that was a critical component of those surveys was a focus group effort that was specifically targeting immigrants, refugees, and non-majority populations, and trying to find out, here in the Municipality of* [city name]*, what were some of the barriers to services or challenges or issues that they were experiencing secondary to COVID.*
Bringing COVID-19 resources to agricultural workers	*There were also some settings where we knew upfront just based on outbreaks within workers who were here temporarily and primarily spoke Spanish on the east side within agriculture settings. So in those cases it was a little bit more of a known quantity that we did need to have some good language capacity available.*
	Theme: cultivating relationships
Partnering with community health boards to build community capacity	*We’re very lucky to have ethnic community health boards in Washington…there’s a coalition of the health boards as well, and I think folks have seen this as a really helpful way for the community to give voice to the things that they are seeing as health needs within their community and too, hopefully, for public health agencies to be able to contribute to that effort.*
Partnering with employers	*In terms of getting hard-to-reach people,* [Company name] *was instrumental with that. Within that company they also had a coordinator…she worked specifically with the employees who were refugees I believe, and so she also helped, coordinate with IRC to make sure that we, had what we needed and, if we needed help with any investigations, she would kind of help…*
Partnerships with state refugee health coordinators	*It’s really important to have the connection with the local health departments and make sure that they feel like their needs are being met and that their concerns are being addressed…on the whole COVID response team…we have a liaison to local health departments. There’s one individual person that heads that up and there’s a group of people, so they work hard at trying to listen to what the locals have to say and what their concerns are. Otherwise, we know that it would not be successful, and it is, ultimately, their jurisdiction.*
	Theme: ensuring cultural and linguistic concordance
Collection of data about language	*…Having language data even reported on the front end of receiving the case is so important in order to make that match between a case investigator and a patient. I think that traditionally public health reporting systems do not always receive that information just through the ways that the channels have systematically been built and the data that’s populated, and even the way that clinicians collect that information on the front end, all those pieces have to go right, and as you are doing a rapid response, that’s one of the areas that I really recognized in the course of this that we need to strengthen…*
Staffing agencies to increase language capacity for CICT	*People really do appreciate getting a phone call from somebody who speaks their language…So that was one thing that was very nice about bringing on a staffing agency is that we were able to get…a broad spectrum of different languages that were spoken throughout*.
	Theme: intentional communication
Utilizing WhatsApp to reach RIM community members before CICT calls	*I used those apps quite a bit so—and one of them is-is the WhatsApp app and then basically I would text and say*, *“This is to help the department and I need to, um, speak – I need somebody to call me back and whatnot.” So it is kind of like I think that it was just—oh, it is a real person* versus *I am just not gonna, um, answer any random calls…*
Cultural Navigators as liaisons between community and public health	*One of the CBO’s* [community based organizations] *that we were working with for this pilot project for cultural navigation…They had rotating on call system where people could call in. There would always be somebody who could answer or call right back to understand if somebody had a question about COVID…Supported by trusted community members.*
Outreach to RIM communities prior to CICT engagement	*I think in addition to the case and contact investigation, part of it too was just the general messaging to communities about “If you get COVID, this is how things will go. Please do pick up the phone. This is why they are asking questions. No, they are not going to ask about your immigration status, or report something back to ICE* [Immigration and Customs Enforcement]*.” Those parallel messages, setting expectations were really, really key.*
	Theme: implementing equity
Embedding equity	*So, again, with COVID and how it’s impacted is, they have developed more—basically, more events where they are giving out food, and then again, over the phone assistance for people who are affected by COVID to kinda give them a priority in regards to assistance financially, because we do have, like, Action Committee,* [name redacted]*, where they help with gas, electricity and water. There’s another—there’s a lawyer firm that actually helps with evictions that we are aware of, that we share their contact information. That way, if someone is in fear of eviction because their home keeps going back into quarantine and positives keep coming…*
COVID-19 community coordinators	*…The [City] based resource coordinators worked with the district resource coordinators and once we found out someone is positive, they cannot go to work, they have got bills they have got to pay, the ball would start to roll and so the contact tracing was now different because the way the state had it structured was you did everything on the phone with ours, we actually went knocking door to door.*
	Theme: evolving responses
Designated CICT trainer and training developer	*…We did not really have a training structure built…I train folks how to conduct interviews, how to conduct interviews, contract tracer calls to people that have tested positive for the virus, or the contacts of those people that have tested positive for the virus…I do a lot of the training development material…A lot of those issue that are very context-needed, that they* [case investigators] *need to know the context of where these people work, how do they work, how do they make a living…And as a bilingual [person] you need to be very sensitive to those things, and very cognizant that they exist. These challenges are very real for them.*
Supporting mental health for RIM communities	*Before we end the call* [case investigation interview]*, of course, we offer assistance and referrals and then assistance in any questions or concerns they may have and fears as well as offer information for mental health.*

* The theme under which the promising practice is located/positioned was selected as a primary theme, however, many promising practices and excerpts span multiple themes.

### 3.3. Understanding community and public health context

Interviewees described that public health organizations at the local and state level utilized their knowledge of community and public health system assets, needs, and challenges, leveraging existing resources to support a comprehensive CICT response to COVID-19.

#### 3.3.1. Community context

Early in the COVID-19 pandemic, state and local public health organizations had a range of understanding around community needs, existing strengths, and resources, including whether or not their public health teams were reflective of RIM communities within their broader community. Interviewees acknowledged the myriad challenges RIM communities faced specific to participating in comprehensive aspects of CICT including: access to and/or fear of testing, the consequences of testing positive when living in high density housing, financial hardships, inability to access information due to language, literacy or technological barriers, and fear of sharing contact information related to potential immigration concerns. Interviewees also frequently identified specific RIM community strengths and resources including community-based organizations (CBOs), multilingual and multicultural media, places of worship, and employers who were developing messaging and/or programs for their employees.

#### 3.3.2. Public health context

Interviewees highlighted the importance of specific public health teams and team members with pre-COVID-19 work experience that provided them a strong understanding of RIM communities in their geographic area, and engagement with partnerships that informed their understanding of community context (e.g., public health nurses assigned to visit community health centers semi-annually for technical assistance). Interviewees also recognized system-level tensions as vital pivot points to support the evolving response and movement toward promising CICT practices (e.g., hiring RIM community members to address CICT staffing-related challenges, or adapting quarantine and isolation resources for a state context when existing resources were culturally, and locality specific). The combination of understanding the community and public health context provided a foundation for adaptation, innovation, and growth for comprehensive CICT for COVID-19 inclusive of RIM communities.

### 3.4. Cultivating relationships

Effective CICT is facilitated by cultivating existing and new relationships that are grounded in trust between public health and RIM communities at the individual, community, and organizational levels. Trusting relationships fostered by individual public health professionals with RIM communities sometimes supported CICT efforts when public health organizations did not have established relationships:

*…I used to direct the migrant farmworker program. So I have a close relationship with that program. I've become the direct link between the farm worker program and the health department…[when] we have an outbreak those cases all come through me…*

In some locations, public health teams already focusing on RIM communities were able to quickly build upon those community relationships, and those with strong established community-public health relationships could be further leveraged in the context of COVID-19:

*That group is a fantastic community-based organization and a known trusted partner, and so when now COVID-19 is the new thing coming in, it was relatively easy to contract with them to provide information to say, "Hey, can you please ramp this up?”*

Interviewees frequently reflected on the importance of organizations fostering trust to facilitate CICT, identifying trust as fundamental to cultivating relationships. They acknowledged the importance of repairing broken trust, bolstering existing trust, and intentionally building trust to facilitate CICT. To foster trust in the CICT process some public health organizations engaged community leaders in case investigation; yet, this was not seen as a way to facilitate trust in every context. One interviewee reported feedback from a community partner indicating,

*folks don't feel comfortable serving in that role [CICT]. They want to maintain their positions of trust in the community and feel like it moves them a little bit into that government role too much if they actually are the ones collecting sensitive information.*

The importance of building sustainable relationships between public health and RIM communities *before* they are needed in a fast-moving environment—like a pandemic response or another emergency—was highlighted across the interviews and well stated by an interviewee:

*It's hard to build a relationship in an emergency … what are the ways that we could support and also structure so that the next time, or even going into the fall now, how can we better support both community and the folks that are doing public health work?*

Importantly, the aforementioned examples illustrate how modifying existing or building new relationships facilitates trust and requires an ongoing focus on communication and understanding community contexts and preferences.

### 3.5. Ensuring linguistic and cultural concordance

Ensuring linguistically and culturally concordant communication, services and support is essential to successful CICT with RIM communities. Interviewees explained that few systems had processes in place to identify languages spoken and/or preferred by COVID-19 positive community members they were attempting to engage in CICT. This gap in knowledge about language for CICT was due to multiple barriers such as language not being collected at the time of testing, or because language information did not have a field in their CICT software. These barriers frequently led to a best-guess approach to spoken language based on name or country of origin, if known. *“[I]t’s an imperfect system but it’s kind of the best that we can work with at this time.”*

Interviewees described operational adjustments during the early phases of the pandemic that allowed multilingual staff members to temporarily shift into roles where they were using their language skills to conduct interviews, or train others on how to use telephonic, video and in-person interpreter services. Eventually organizations shifted to hiring practices that intentionally sought individuals with community-matched language skills and cultural backgrounds, or contracting with staffing organizations with multilingual staff. Interviewees also highlighted the nuance between people who speak a language as their heritage (primary) language compared to non-heritage speakers, observing a stronger sense of community trust with people who were heritage speakers. Herein lay the importance of both linguistic concordance—speaking the same language as the person you are interviewing—and cultural concordance—understanding cultural relevance, preferences, variations in language, and a shared framework of expectations.

The interviewees also recognized the need to ensure health education materials were available in the language and form of media that is preferable and accessible to the audience (e.g., oral PSAs or videos might be preferable to written material). Interviewees highlighted the importance of understanding and reflecting on the breadth of languages spoken within their jurisdiction (a point of intersection with the *Understanding Community and Public Health Context theme*), and the importance of prioritizing the development and dissemination of linguistically and culturally relevant materials, especially given the gap could lead to inequity in information access for smaller and less common linguistic communities. *“The weekly briefs from the mayor, they are always in English, so how do they get translated down to other languages … that real-time feedback from your mayor or leader during an emergency, that’s really tough.”*

### 3.6. Communicating intentionally

Intentional communication between public health organizations and RIM communities facilitated sharing and receiving information and promoted engagement in the CICT process. Interviewees described that public health organizations were messengers about CICT both at the individual level during CICT interactions, and at the community level disseminating information about COVID-19 and CICT. CICT interactions for COVID-19 needed to involve building trust with the person being investigated while simultaneously offering CICT process guidance, collecting information, providing COVID-19-specific resources, addressing concerns, and giving accurate messages with an emphasis on promoting community and individual health. As one interviewee described, *“That care coordination piece in addition to* [the] *public health piece about ‘Here’s the things you have to do’, those two things in tandem are pretty important.”*

Individually, interviewees described providing public health guidance in a systematic and factual manner as part of the CICT process, *“sure, we use scripts. But we are not salespeople … We’re not trying to sell something. We’re not trying to get them to do something. We’re trying to provide public health guidance.”* One trust-building communication strategy shared by an interviewee was intentionally framing the request for information that accompanies the initial CICT phone call as information that has the potential to benefit that individual, their family and the health of the community. Several interviewees described that after years of being advised not to share their personal information, RIM community members being contacted by public health practitioners asking for personal information over the phone was understandably not well received. Consequently, the need to effectively message the function and significance of CICT at the community level, via trusted community sources, was critical to successful CICT.

Additionally, iterative communication was needed within and between CBOs, employers, and public health to ensure everyone had up to date information; however, interviewees highlighted the challenges of making time for these “check-ins” during the pandemic and the “bureaucracy” that made getting the message out from public health slower than planned. As with many of the system-level tensions that became apparent or exacerbated during COVID-19, these became pivot points for public health as the response evolved. For example one interviewee described an appreciation for and recognition of the places where communities might be able to spread and receive messages more quickly than traditional public health messaging such as faith-based organizations: “*The* [redacted] *Community Center is not faith-based but does work with a lot of the different churches and has built a lot of relationships and rapport over time. Their director actually recorded a message about COVID-19 and health education … So the church leaders played it on Sunday during their Zoom services across – to disseminate information from this single place and trusted source.”* Other interviewees also emphasized the value of public health getting messages out via social media in partnership with “*community level influencers and CBOs and then also being receptive to the feedback, creating that dialogue and being receptive to the feedback*.”

### 3.7. Evolving response

The approach to CICT evolved over the COVID-19 response and required ongoing adjustment of public health roles, processes, and infrastructure to address organizational and community needs. Public health professionals experienced tension between stressed resources and the ability to innovate at the pace and in the ways that they wanted to for effective and comprehensive CICT. Organizations were unable to provide CICT for every person, and in many scenarios needed to prioritize case investigation, leaving contact notification to individual cases. Many interviewees described the urgent needs and emerging challenges RIM community members shared as the response evolved (e.g., feeling unsafe in their home, or having concerns of job loss while in quarantine and isolation). These challenges led to the development of approaches to support people through the CICT process such as explaining quarantine and isolation in detailed and culturally relevant ways, while also identifying financial or food resources, housing, or other supportive services.

To meet staffing needs, public health professionals utilized their knowledge of existing resources to identify other roles within their system (i.e., navigators or people who conduct CICT for tuberculosis), partner with other organizations (i.e., students at universities), and/or hire from contract agencies and recruited people from retirement. Ideally, public health organizations would have preferred to hire more staff from RIM communities; however, they described challenges finding time to navigate recruitment and hiring policy and procedure challenges, especially early in the pandemic. Public health organizations needed to adapt when engaging with workers in seasonal industries such as farms, food packing, and fishing, for example how to provide housing if an outbreak happened in communal housing. The ongoing evolution of the response also meant public health employees frequently shifted roles. Interviewees spoke about the energy and time their organizations required to continuously shift resources and staff, which contributed to staff stress and burnout, as described by one interviewee, *“…the public health folks are exhausted. Everybody’s exhausted. It’s been a year, and people are tired, and now we are trying to get vaccine out as fast as possible*.” Similarly, another interviewee states*“It’s the most intense professionally and personally [I have worked in] my life … we need to learn to adjust to this new reality, and that does not just include the health department … all of healthcare and all of society for that matter.”*

### 3.8. Implementing equity

Public health systems varied in their emphasis on equity in implementing comprehensive CICT and supportive processes. Notably, this theme was not directly asked about in the interview guide, however, all of the interviews describe awareness of equity or inequity, and varying levels of equity in action. Within some public health organizations, interviewees identified specific people and teams who were focused on health and/or language equity that were trying to improve and enhance communication with immigrant communities. Interviewees also identified structural factors within the broader public health COVID-19 response that were contributing to inequities in data collection, testing and vaccine distribution, and characteristics of leadership and team structures. Collecting language at the time of testing was highlighted as a key area where health departments should improve their data collection. One interviewee explained

*“… we realized that we were not capturing the data that we really needed, all the way down to the languages, to really be able to make strong recommendations to the developers of the software, to the developers of the contracted labs that we were using. So we began to take a deeper dive with that and then the governor started making executive orders to say to the labs, ‘you’re now required to collect race, ethnicity and language.”*

Interviewees provided several examples of missed opportunities for language collection at the time of testing potentially contributing to their inability to identify individuals and communities who were being disproportionately impacted by COVID-19 infection, but underrepresented in the data because it was not being collected. Additionally, access to testing was limited for some communities because it was only done at drive up sites, making it inaccessible to anyone without a car.

Interviewees identified key areas where they sought support both outside and within their organizations to ensure equitable support for immigrant communities. In settings where employers provided housing such as for seasonal work, public health organizations played active roles in identifying sites for quarantine and isolation, and supporting on site testing and vaccination. Interviewees described that many people were facing challenges with meeting basic needs due to loss of wages, jobs or missing work due to illness. One interviewee emphasized, “*Assuring that the distribution of those social supports and financial resources was equitable, I think, is a really big challenge, and one of the challenges for folks accessing that support was language on the phone when they called for help.*” A few public health professionals noted that teams that met weekly to debrief, reflect, and learn from one another were well positioned to identify equity concerns and raise them to leadership. Interviewees highlighted that training about discrimination, bias and cultural humility was important to be effective in comprehensive CICT and all public health activities.

Some interviewees also described the need to engage in advocacy within their organization to ensure funding could be allocated to CBOs. In many cases CBOs focused on communities disproportionately impacted by COVID-19 were doing the bulk of the public health work without a commensurate amount of funding. To address this barrier, some public health organizations sought creative ways to support partnerships, including one example of funding a health equity center led by a CBO and an academic medical center:

*…our state was able to allocate funding specifically to provide COVID-19 education to communities that were disproportionately impacted by COVID-19 and so they did that relatively early on and pushed money to community-based organizations all around the state to do that direct outreach and support within their communities.*

The continuum of equity awareness and implementation varied across public health, indicating a need for an ongoing, multilevel support for teams and systems to expand equity efforts.

## 4. Discussion

Professionals working at public health organizations identified six themes that facilitate CICT for COVID-19 with refugee, immigrant and migrant communities: understanding community and public health contexts, cultivating relationships, ensuring linguistic and cultural concordance, communicating effectively and intentionally, acknowledging the evolving response, and implementing approaches with equity. There were many barriers discussed to CICT with RIM communities in the results, however, we chose to focus more on facilitators and promising practices in the discussion in order to elevate the positive deviants and lessons for future responses.

These themes and identified promising practices (Table 2) learned and re-learned by public health organizations during COVID-19 for CICT with RIM communities can be translated to other areas of public health practice that involve CICT or benefit from lessons learned herein: sexually transmitted infections, monkeypox, tuberculosis, vaccine preventable infections, foodborne illness, non-communicable diseases, lead exposure and injury prevention, and more. We paired the themes from this analysis with action items and resources to operationalize comprehensive CICT with refugee, immigrant and migrant communities (Table 3). The action items are derived from the public health professional interview data in our analysis and intended to facilitate comprehensive CICT. The action steps are organized with additional resources identified by our team to provide practical steps for readers.

**Table 3:** Action steps and resource, organized by theme,^*^ for operationalizing comprehensive COVID-19 case investigation and contact tracing with refugee, immigrant, and migrant communities (data from multiple United States HHS regions; September 2020–February 2021).

Understanding community and public health context: use a layered approach to understanding community and public health system context to inform understanding and interventions.	
Action steps	*Evaluate public health and community context while considering current and historic relationships and partnerships.* *Community context:* build on existing resources and understanding of RIM communities in your area through a rapid community assessment. RIM communities’ countries of origin. Languages spoken. Geographical location/housing. Established partnerships with community organizations. Health providers. Schools. Employment/industry. *Public health system context:* evaluate the existing resources within the public health organization to support RIM communities. Identify specific teams within the organization focused on RIM communities. Evaluate staff demographics and alignment with RIM communities. Incorporate multilingual and RIM communities within emergency response structure. Access to scale up language services. Enhance data collection of language on surveys, testing, when services are accessed. Review policies to ensure that newly developed and existing materials are translated and culturally reviewed. Consider approaches to funding community organizations and scaling up language services through staffing, contracting, or partnering. Conduct, update, and review annually a rapid community assessment.
Resources	NRC-RIM guiding principles. How to conduct a rapid community assessment and the addendum: considerations for conducting community assessment with refugee, immigrant, and migrant communities. Community mapping NACCHO Health Equity and Social Justice Initiative and Resources Designing and implementing equitable and inclusive Survey or NACCHO Public Health Quality Improvement Resources
Cultivating relationships: facilitate CICT by building on existing relationships and cultivating new relationships between public health and RIM communities.	
Action Steps	*Prioritize trust-building* Understand the community’s current and historically contextualized story/experience of trust and/or distrust with systems. Engage in trust-building activities at the: Individual /staff level Personal awareness of bias. Cultural responsiveness training. Awareness of SDOHS impacting local immigrant communities. Public health organization level Policies and practices that build trust within communities. Supportive staff training. Hiring practices that include immigrant community representation.
	*Consider characteristics of building new and strengthening existing relationships, and consider creating an inventory, which may include the following areas:* Temporality (i.e., location on the continuum of new, existing, long-term relationships). Level (e.g., individual, program or organizational level) and consider if policies are in place to support relationships at these levels Category of relationship (e.g., informal or formal, community and/or organizational entities such as immigrant centered non-profit organizations, faith organizations, and employers). Trajectory (e.g., mutually agreed upon goals and MOUs such as a set type of work to be done under contract, short-term/circumstance-dependent staffing support, or a sustainable/long-term partnership with multi-faceted goals).
Resources	Case investigation and contact tracing: build trust with targeted strategies. Partnerships: NRC-RIM guides, checklists and promising practices. Community engagement toolkit (with guides, checklists, and promising practices). Building partnerships across communities and systems. Benefits of community advisory boards. Community health workers. Partnering with FQHCs serving RIM communities.
Communicating intentionally: engaging in intentional communication with immigrant communities facilitates sharing and receiving information and promoting engagement in CICT.	
Action Steps	*Assess existing communication structures and processes, facilitators and barriers* Inventory existing: Communication channels. Messages. Trusted messengers. Technological facilitators (e.g., WhatsApp thread). Identify communication challenges in the inventory at the community and public health organization level.
	*Review methods and approaches to bi-directional flow of information both listening and sharing* *Listening:* Consider ways the organization is listening and learning information from the community, including: Surveys. Focus groups or listening sessions. Community conversations. Written or voice feedback. Other community-informed listening strategies. *Sharing:* Consider these components of impactful messaging from the organization to the community. Audience (e.g., individual, family, specific community, and community organization). Approach (e.g., authentic/genuine, personalized, empathetic, and declarative). Message clarity (e.g., general or COVID-19 specific health education). Messenger (e.g., trusted messengers such as community or faith leaders, culturally and linguistically concordant staff). Channel/method (e.g., in-person, phone call, text message, WhatsApp, translated written materials, social-media, video messaging, radio, print media, technology supported CICT/quarantine. and isolation/health monitoring tools and systems).
	*Communicate intentionally, intra and inter-organizationally* Intra-organizational communication strategies such as data sharing, clearly defined and communicated CICT processes, training development and implementation, and intraorganizational communication tech support. Inter-organizational communication strategies including seeking out/inviting, listening to and elevating/acting on stakeholders’ experiences/knowledge and suggested strategies, making sure to include community partners and public health staff/teams.
Resources	Effective communication guides, checklists, and promising practices. Outreach to RIM communities ahead of CICT efforts. Translation process and translated materials library. Build your own CICT campaign. Working toward equitable language access.
	Ensuring linguistic & cultural concordance: ensure linguistically and culturally concordant communication, services and support for RIM communities.
Action Steps	*Assess existing communication structures and processes, facilitators and barriers specific to linguistic and cultural concordance* Identify what is working well to ensure linguistic and culturally concordant communication. Identify language and culturally concordant/discordant messaging-related challenges Lack of data and/or data sharing indicating preferred language. Lack of access to language services when engaging in CICT (e.g., multilingual/multicultural staff unavailable, language line services unavailable/limited access, and PH unaware of how to work with interpreters/need for training).
	*Operationalize linguistic and cultural concordance as a best practice* Language preference data collection and sharing practices, and identifying the processes needed when the data is not available. Leverage, hire or contract with multilingual/multicultural staff whenever possible. Provide opportunities for ongoing formal/informal training and learning exchanges around culturally concordant messaging.
	*Consider approaches to linguistic and cultural concordance within your community and public health context* Assess availability of language services (i.e., not always available for less common languages). Review policies, procedures, and institutional resources to translate and culturally validate new or existing messaging.
	*Explore staffing variations, with a focus on consistency and sustainability* Temporary public health internal re-deployment of language-proficient staff roles to meet RIM community CICT needs. Interpretation line availability. Contract staffing (e.g., companies, CBOs). Partner supported (e.g., CBOs, academic partners, and volunteers). Hiring new staff, ideally from local communities.
	*Evaluate multilingual methods/channels* Call-in-lines (through CBOs, health systems, public health). Text messaging. Communication systems (e.g., Sara Alert). Audio and video PSAs. Social media posts.
Resources	Cultural navigators and/or Staffing agencies to increase language capacity. Multilingual coffee hour peer support group for CICT teams. Tips for working with interpreters during CICT. Working toward equitable language access. Sara Alert to automate symptom monitoring. Language access and content validation. Working with Interpreters during CICT.
Evolving responses: track and reflect on how emergency response CICT practices evolve over time to address community and system-level needs, challenges, new information, and changing public health guidance, as a way to enhance current and future responses and outcomes.	
Action Steps	*Monitor response evolution over time* Recognize *early* stage response needs. Consider approaches that facilitate adapting to frequent changes in guidance. Prioritize additional staffing and training based on the response needs over time.
	*Responding to a new public health need requires ongoing inventory of challenges, and approaches for adaptation and innovation* Ensure data sharing with attention to confidentiality Address funding barriers (i.e., resources and support services for cases and contacts in the context of CICT). Consider legal and economic/employment related issues for cases and contacts. Address the absence of necessary policies, procedures, or financial mechanisms to allow for rapid movement of funds and approaches. Navigate high staffing needs while recognizing staffing burnout.
Resources	Continuing education: learning through training and NRC-RIM webinars. Social support services for RIM communities: a checklist for health departments. Supporting mental health in RIM communities during COVID-19 and beyond. Psychological first aid for CICT staff.
Implementing equity: creating processes that acknowledge and implement equity from within the public health system.	
Action Steps	*Need to develop and implement processes to re-evaluate activities with an equity lens*. Evaluate programs, processes and policies with a formal tool, such as the health equity impact assessment tool. Proactively promote programs and activities developed with an equity lens. Ex. Pre-COVID activities such as a local public health consortium developing broad strategies to serve at-risk populations. Ex. Intentional development of collaborative approaches in partnership with communities (i.e., vaccine rollout).
Resources	Creating health equity zones. Embedding equity throughout the COVID-19 response. COVID-19 vaccine collaborative. Conduct a health equity impact assessment. Equity is fundamental to implementation science.

* This table presents data-informed action steps organized by primary theme, accompanied by resources selected by the NRC-RIM team that may aid in operationalizing comprehensive CICT for RIM communities. Many of the action items and additional resources provided are applicable to current and future comprehensive approaches to CICT, and other public health strategies/practices, expanding beyond COVID.

**Figure 1:**
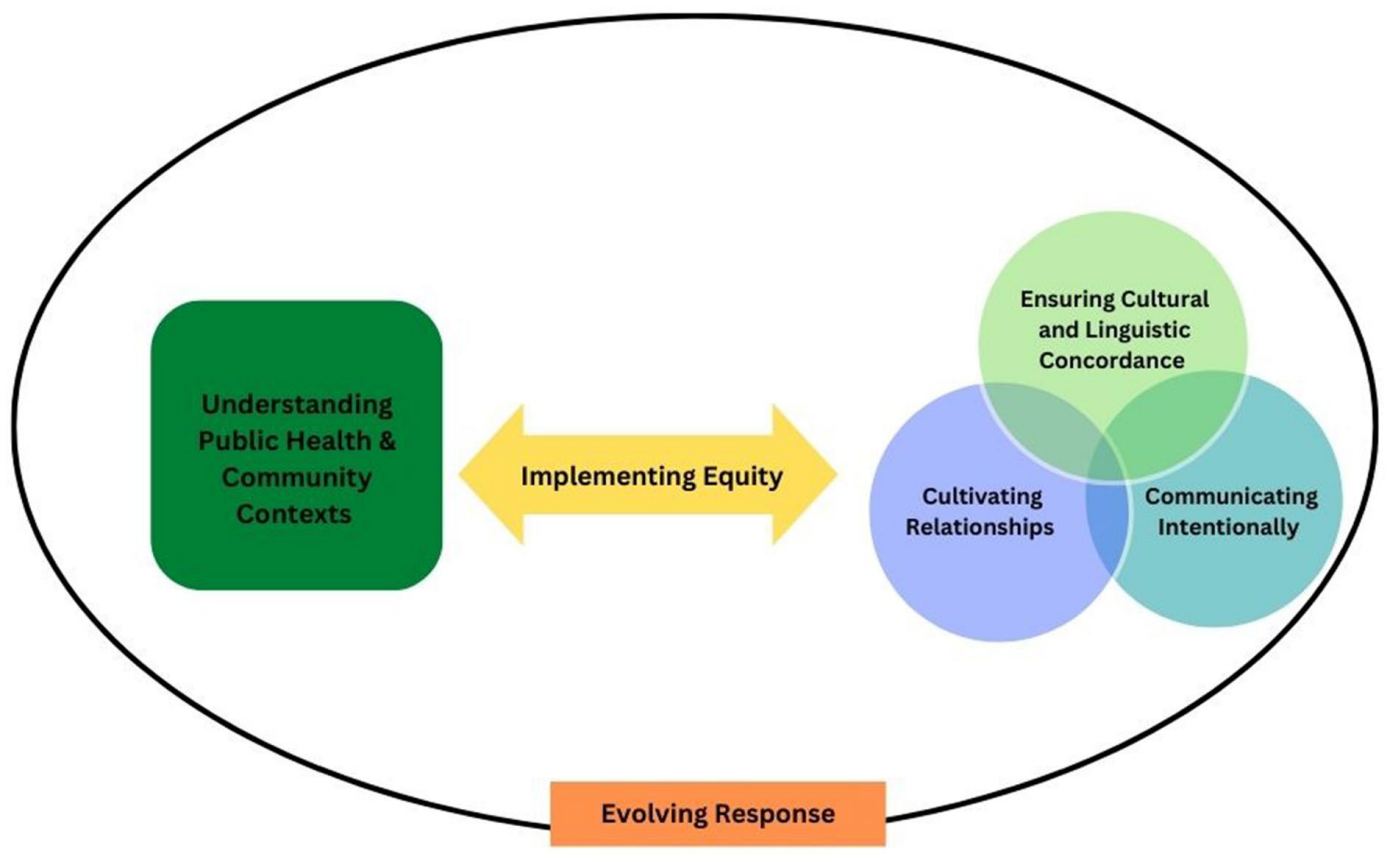
Comprehensive COVID-19 CICT promising practice facilitators: A thematic map of public health professional perspectives from the U.S. HHS Regions from September 2020 to February 2021. Understanding community and public health contacts stands on its own as a square (representing a foundation) as a facilitator of CICT. The three themes of: (1) cultivating relationships, (2) ensuring cultural and linguistic concordance; and (3) communicating effectively and intentionally each stand alone in their circles but also share key common features and often operate together. The un he understanding community and public health context square and the three intersecting circles are connected through a bidirectional arrow of Implementing equity. We believe that the interaction between these four themes are critical to organizations being able to develop and implement programming addressing disparities toward a goal of equity. Collectively, all five of these themes are operating within the six theme of the evolving response to the COVID-10 pandemic, therefore, the evolving response is represented as a circle around all themes.

In order to describe visually how the six themes may inform future CICT or other public health responses we interpreted the relationship between the six themes in Figure 1. Understanding community and public health contexts stands on its own as a square (representing a foundation) as a facilitator of CICT. The three themes of: (1) cultivating relationships, (2) ensuring cultural and linguistic concordance, and (3) communicating effectively and intentionally, each stand alone in their circles but also share key common features and often operate together therefore they overlap with one another. The understanding community and public health context square and the three intersecting circles are connected through a bidirectional arrow of implementing equity. We believe that the interaction between these four themes are critical to organizations being able to develop and implement programming to address disparities and move toward a goal of equity in health care outcomes Collectively all five of these themes are operating within the sixth theme of the evolving response to the COVID-19 pandemic, therefore, the evolving response is represented as a circle around all themes. Acknowledgement of the way these themes may operate together can inform community-centered CICT and other public health response activities.

The World Health Organization (WHO) released an *Operational Guide for Engaging Communities in Contact Tracing* of “best practice principles for community engagement and how they can be operationalized as part of any community-centered contact tracing strategy” (23). It includes 11 key principles: (1) understanding community context, (2) build trust, (3) ensure and maintain community buy-in, (4) work through community based solutions, (5) generate a community workforce, (6) commit to honest and inclusive two-way communication, (7) listen, analyze, and respond to feedback, (8) consider the use of contact tracing technology, (9) do not criminalize actions, (10) discourage and address, and (11) coordinate with all response actors. These WHO key principles closely align with the themes we identified with public health professionals in the US working with refugee, immigrant and migrant communities, during COVID-19 in 2020–2021, particularly key principles 1–7.

The WHO guidance describes the important role of people who are migrating or have experienced migration as key to include in developing community-centered CICT (23). The WHO guidance included limited reference to community-centered approaches in non-dominant languages (23). Therefore the findings of our thematic analysis about the importance of linguistic and cultural concordance, and concrete promising practice examples, provide an additional area for emphasis to support all communities in CICT. Teams that are more reflective of the communities they partner with noted that their cultural and linguistic concordance helped people of similar identities be more comfortable engaging in CICT (12, 16). Public health organizations that collected data about language at the time of COVID-19 testing matched linguistically concordant staff members with the case or contact, emphasizing the importance of routine language collection for public health surveillance (24). Often sustained partnerships between public health and CBOs led by refugee, immigrant and migrant communities played key roles in linguistic and cultural support. For example, a longstanding, 17-year community-engaged research partnership in southeast Minnesota quickly adopted a crisis and emergency risk communication framework in 2020 to address COVID-19 and reached 39,000 people in seven languages over a 6 month period with community-led COVID-19 messaging (25).

As the COVID-19 pandemic unfolded, public health professionals and organizations needed time and space to be creative and innovative, and policies that supported this innovation. Frequently, however, the resources and policies needed to support innovation were not available. As public health continues to learn from COVID-19, a sustained investment in public health system strengthening as an element of pandemic preparedness that includes refugees, immigrant and migrants will foster proactive solutions beneficial both for the public health crisis at-hand and for staff well-being. Public health system strengthening for pandemic preparedness and surge capacity planning could include: (1) enhancing epidemiologic surveillance including routine collection of language, race, ethnicity, and nativity data to inform public health interventions and predict workforce needs (24); (2) identifying funding mechanisms, resources and relationships that can be leveraged quickly in an emergency for surge capacity such as the ability to pay CBOs working in partnership with public health organizations; and (3) developing strategies to both retain and also to quickly onboard public health staff with linguistic and cultural expertise to ensure that language equity is a core component of any evolving response; and (4) funding program evaluation and research focused on CICT and other public health interventions with RIM communities. In sum, creating and sustaining mechanisms for linguistically-and culturally-informed community-engagement should be integrated into public health system strengthening and preparation for the next pandemic.

### 4.1. Limitations

The interviewees in this analysis were from professionals working within public health organizations, therefore the identified perspectives are from this vantage point and may be different than recommendations from community based organizations or health systems. These perspectives were described elsewhere (16, 17). The interviewees who participated had the bandwidth to participate in an interview during the early part of the COVID-19 response, and may not represent the perspectives of significantly burdened systems. Additionally, though findings and dissemination materials from this project might not be generalizable beyond the scope of this project, they might be transferable in some contexts/populations, with consideration of the project limitations.

## 5. Conclusion and public health implications

The thematic findings and promising practices identified in this project support proactive, community-engaged solutions for public health and other organizations working and partnering with refugee, immigrant, and migrant communities. Implementing these findings with COVID-19 into current and future public health responses could improve public health, language equity, collaborations with refugee, immigrant and migrant communities, and staff wellbeing. Lessons learned from comprehensive CICT with RIM communities and sustained investment in public health system strengthening may facilitate equitable implementation (26) through community-engaged responses across all public health programming.

## Data availability statement

The raw data supporting the conclusions of this article will be made available by the authors, without undue reservation.

## Author contributions

ED-H conceived and supervised the study and led the writing. WF led the analysis and co-wrote the manuscript. SK, SA, DA, YG, SH, and CT assisted with the study and analyses. FM assisted with the analyses and the writing. SE developed the figure and contributed to the writing. MK assisted with the study, interpretation, and writing. KaY supervised the study, and contributed to the writing. All authors contributed to the article and approved the submitted version.

## Funding

This study was supported in part by the National Resource Center for Refugees, Immigrants, and Migrants which is funded by the US Centers for Disease. Control and Prevention and the International Organization for Migration (award number CK000495-03-00/ES1874) to support health departments and community organizations working with Refugee, Immigrant, and Migrant communities that have been disproportionately affected by COVID-19. SA received support from the University of Washington National Research Service Award—Child Health Equity Research Program for Post-doctoral Trainees (T32 HD101397). CT received support from the National Institute of Allergy and Infectious Diseases of the National Institutes of Health through the University of Minnesota T32 award (T32 AI055433). This study utilized REDCap electronic data capture tools which was funded by Institute of Translational Health Science (ITHS) grant support (UL1 TR002319, KL2 TR002317, and TL1 TR002318 from NCATS/NIH).

## Conflict of interest

The authors declare that the research was conducted in the absence of any commercial or financial relationships that could be construed as a potential conflict of interest.

## Publisher’s note

All claims expressed in this article are solely those of the authors and do not necessarily represent those of their affiliated organizations, or those of the publisher, the editors and the reviewers. Any product that may be evaluated in this article, or claim that may be made by its manufacturer, is not guaranteed or endorsed by the publisher.
