# Supplementary material for: Perspectives of public health organizations partnering with refugee, immigrant, and migrant communities for comprehensive COVID-19 case investigation and contact tracing
Source: Front Public Health. 2023 Sep 5;11:1218306. doi: 10.3389/fpubh.2023.1218306 (PMC10508845; doi:10.3389/fpubh.2023.1218306)
Supplement: Supplementary file 1 [file Table_2.DOCX]

Perspectives of Public Health Organizations Partnering with Refugee, Immigrant and Migrant Communities for Comprehensive COVID-19 Case Investigation and Contact Tracing

**Authors:**

Elizabeth Dawson-Hahn^1,2*^, Windy Fredkove^2,3^, Sayyeda Karim^2^, Farah Mohamed^4^, Seja Abudiab^1^, Diego de Acosta^3^, Sabrina Ebengho^1^, Yesenia Garcia^5^, Sarah Hoffman^2,3^, Megan Keaveney^6^, Erin Mann^2^, Christine Thomas^2,7^, Kimberly Yu^3^, Katherine Yun^8^

***Correspondence:**

Elizabeth Dawson-Hahn

[eedh@uw.edu](mailto:eedh@uw.edu)

**Supplemental Table 1. Semi-structured interview guide with public health organizations about case investigation and contact tracing (CICT) with refugee, immigrant and migrant (RIM) communities**

| 1. Please describe your position.      1. Can you describe the demographics of RIM communities in your health jurisdiction?      1. Please describe your case investigation and contact tracing program.      1. The next series of questions are about the availability and use of language interpretation services to facilitate case investigations and contact tracing efforts with RIM populations.    1. When and How are the language needs/preferred language(s) of the clients determined?    2. Are case investigators/contact tracers available to conduct interviews in those languages?    3. Are interpreters available? If yes, how are they accessed?    4. Are community health workers, cultural navigators or patient navigators a part of the process?      1. Please describe how the case investigations with RIM populations work.      1. Please describe how the contact tracing with RIM populations works. 2. Please describe testing access in your area. What is the process for contacts to get tested?      1. Are contacts monitored daily during quarantine or isolation?    1. If so, what methods are used?    2. If RIM contacts are not monitored daily, please describe any barriers.    3. Please describe the guidance given to cases and contacts around quarantine and isolation. 2. What support and information are provided to cases and contacts after the initial discussion? 3. Is there a community support network for conducting case investigations and contact tracing? 4. What training do case investigators and contact tracers have before they begin? 5. What methods has your jurisdiction used to educate RIM communities about contact tracing?      1. What tools and materials have been useful to your public health jurisdiction in case investigation and contact tracing in partnership with RIM communities? 2. What other successes has your health jurisdiction had with case investigations and contact tracing that we have not yet discussed? 3. What other challenges or system gaps has your health jurisdiction had with case investigations and contact tracing that we have not yet discussed? 4. Do you have any suggestions for how to improve case investigations and contact tracing? 5. Have you been involved in COVID-19 vaccination planning? 6. Has there been any discussion of COVID-19 vaccination in RIM communities? 7. Do you have any materials or tools (websites, data collection tools, etc) that you would like to share with us related to our conversation today? 8. What additional information would you like to share about case investigation and contact tracing, potential future vaccination or the overall COVID response? |
| --- |
